# Digitally designed guided implant surgery in deficient maxillary ridges: Case reports

**DOI:** 10.34172/japid.2022.002

**Published:** 2022-02-21

**Authors:** Neethi Deborah Devadason, Senthilkumar S, Rajasekar S

**Affiliations:** ^1^Department of Periodontology and Implantology, Rajah Muthiah Dental College and Hospital, Annamalai University, Chidambaram- 608002, Tamil Nadu, India

**Keywords:** Bone density, computer-aided design, dental implants, osteotomy, 3-D printing

## Abstract

Periodontitis can lead to the loss of hard and soft tissues of the oral cavity. Dental implants have become a reliable treatment modality in recent times, especially with the evolution of digital technology such as CBCT, implant planning software, computer-assisted manufacturing, and guided implant surgery. Documentation of such advancements and their clinical implications would add to the existing knowledge on implant dentistry, encouraging dentists to approach complex implant surgeries confidently. This paper discusses the rehabilitation of missing teeth by applying computer-assisted guided implant placement in two cases with deficient bone volume anteriorly and posteriorly in the maxilla, respectively. Digital planning and careful execution have resulted in precise implant placement and complete osseointegration. In these cases, we could devise treatment plans with both anatomical and prosthetic considerations while being minimally invasive and more predictable, with shorter treatment time and greater patient comfort.

## Introduction

 Implant dentistry has grown by leaps and bounds over the last decade such that it has caught the attention of every clinician. Due to all the technological improvements over the past few years, it may even be safe to claim that implants are now becoming the preferred choice for replacing missing teeth, even in the most difficult conditions.

 Three-dimensional (3D) guided implant placement is one of the recent approaches, where a 3D-printed stereolithographic surgical template is prepared with a computer-aided manufacturing system, with information obtained from the patient’s cone-beam computed tomography (CBCT) images in Digital Imaging and Communications in Medicine (DICOM) format, and from the patient’s working cast models that are scanned, or by using an intraoral scanner directly, using an implant design software.^1,2^

 This report discusses two different cases of guided implant surgery, including the process of diagnosis, treatment planning, patient’s preferences, the procedure, and the outcome. This article was prepared according to the CARE guidelines.^3^

## Case Reports

###  Case 1 

**Figure 1:**
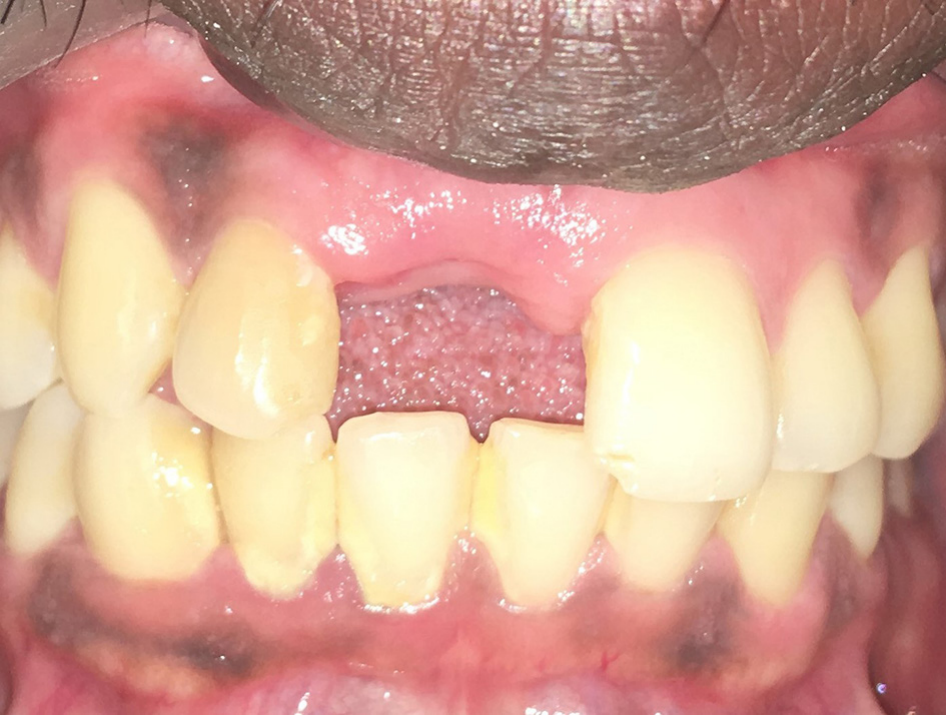
Preoperative photo of the missing tooth (#11) (case 1).

**Figure 2:**
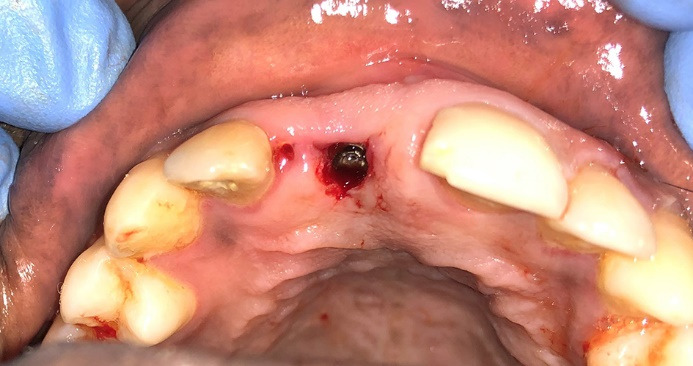
3.8×13 implant placed (#11).

 A 32-year-old male patient reported to the Department of Periodontology for dental implant placement of his missing upper front tooth (Figure 1). The patient was systemically healthy with no adverse habits and had satisfactory oral hygiene and good periodontal health. Following a routine oral prophylaxis procedure, impressions were made, and study and working casts were prepared. The patient was asked to obtain a CBCT (Pax-i3D Green SC; VATECH, Gyeonggi Province, South Korea) to further investigate the remaining bone levels and bone quality. The planning and the surgical procedure were as mentioned in the section below. An implant measuring 3.8×13 mm was planned to replace the missing tooth #11 (Figure 2).

 The DIO IDx (DIO NAVI, Busan, South Korea) device uses resonance frequency analysis (RFA) measurements. Implant stability quotient (ISQ) points >70% were recorded, indicating adequate primary stability. ISQ values indicate the mechanical stability of dental implants obtained by a non-invasive method after surgical placement by resonance frequency measurements.^4^The patient was given postoperative instructions and medications and was reviewed after a week for soft tissue healing and after five months for osseointegration.

###  Case 2

**Figure 3:**
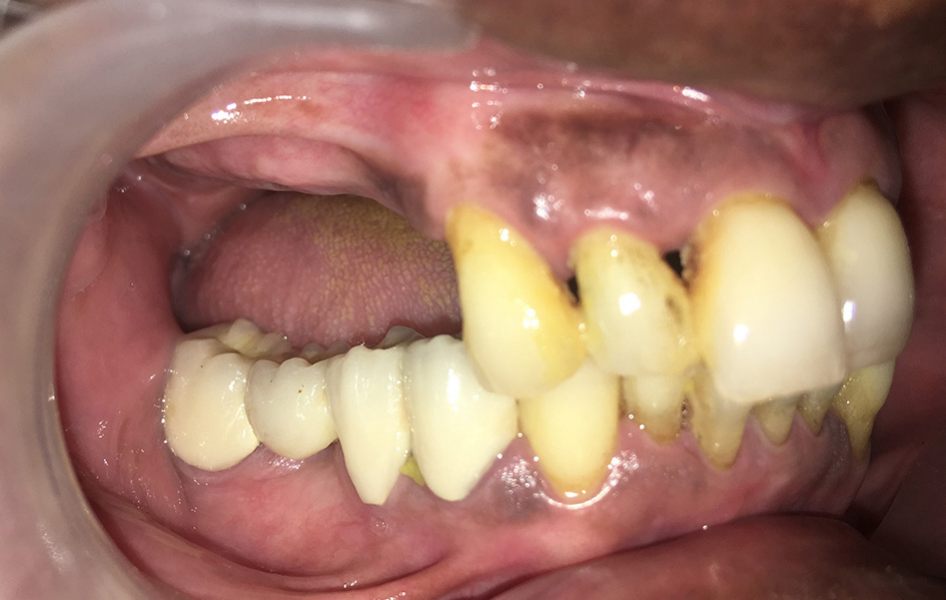
Preoperative photo of missing teeth (#14 to #17) (case 2)

 A 72-year-old male patient with a history of hypertension and osteoarthritis (for which he is undergoing treatment) reported to our department, asking for the implant placement of missing teeth in the upper right posterior region (Figure 3). The patient had fixed partial dentures in the lower arch posteriorly and some root stumps in the second quadrant. This article will only discuss the implant placement planned for his upper right quadrant. The patient also had good oral hygiene status and stable periodontal health. The impressions were taken, model casts were prepared, and the patient was advised to undergo a CBCT (Pax-i3D Green SC; VATECH, Gyeonggi Province, South Korea) imaging procedure. The detailed planning and surgical procedure were as mentioned in the section below.

**Figure 4:**
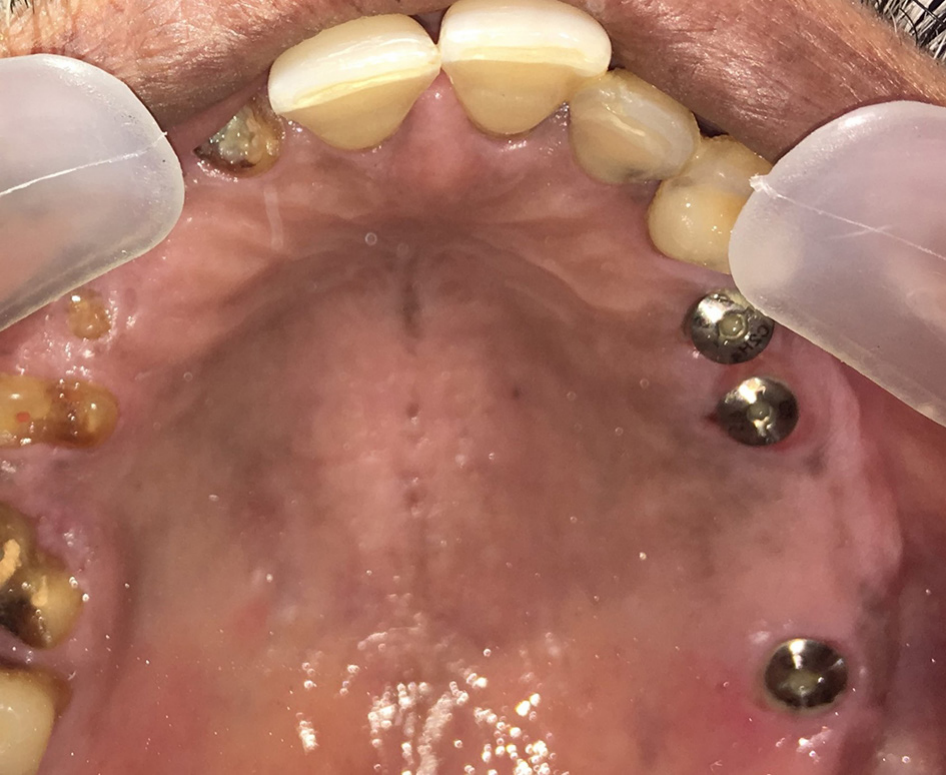
4×10-, 4×8.5- and 4.5×8.5-mm implants placed (#14, #15, and #17, respectively).

 In this case, indirect sinus elevation was required, which was carried out using a balloon technique concerning teeth #14, #15, and #17. Following site preparation, implants (4×10, 4×8.5, and 4.5×8.5 mm, respectively) were placed (Figure 4), and RFA measurements were made using the DIO IDx (DIO NAVI, Busan, South Korea) device, recorded ISQ points of >80%, indicating adequate primary stability. The patient was then given postoperative instructions and medications and was reviewed after a week for soft tissue healing and after 5 months for osseointegration.

###  Planning and surgical phase

 The implant system used in these cases was DIO NAVI (Busan, South Korea). This method is prosthetically driven, i.e., the position of the tooth is first determined before placing the implant. For both cases, patient consent was obtained before implant surgeries. Table 1 indicates the procedural steps in both cases.

**Table 1:** Procedural steps in both cases

**Stages of the treatment procedure**	**Procedural steps**
1. First appointment	• Recording of the case: clinical examination and diagnosis. • Oral prophylaxis procedure • Impressions were made
2. Other investigative procedures	CBCT, routine blood investigations
3. Laboratory procedures	Models scanned
4. Second appointment	• Patients were reviewed; postoperative prophylaxis procedure • The treatment plan was explained, respectively. Benefits and risks were also explained. • The surgical template was checked for adjustments, if any.
5. Third appointment	Surgical phase
6. Fourth appointment	Immediate postoperative review
7. Fifth appointment	The patient was reviewed for healing and osseointegration.
8. Subsequent appointments	• Restorative phase • Follow-up for further review of the implants placed

 In both cases, following clinical examination, impressions were taken, and model casts were prepared and sent to the laboratory for further work. The models were scanned, and the 3D virtual image obtained was combined with the CBCT in the DICOM format to create a 3D view of the jaw and other dental hard and soft tissues, using the software that gives a graphic view of the anatomical structures. The positions of the implants were decided and then virtually placed using the 3Shape implant studio software (3Shape Global; Copenhagen, Denmark). Once the planning and design were over, a 3D-printed template was fabricated by the DIONAVI laboratory.

**Figure 5:**
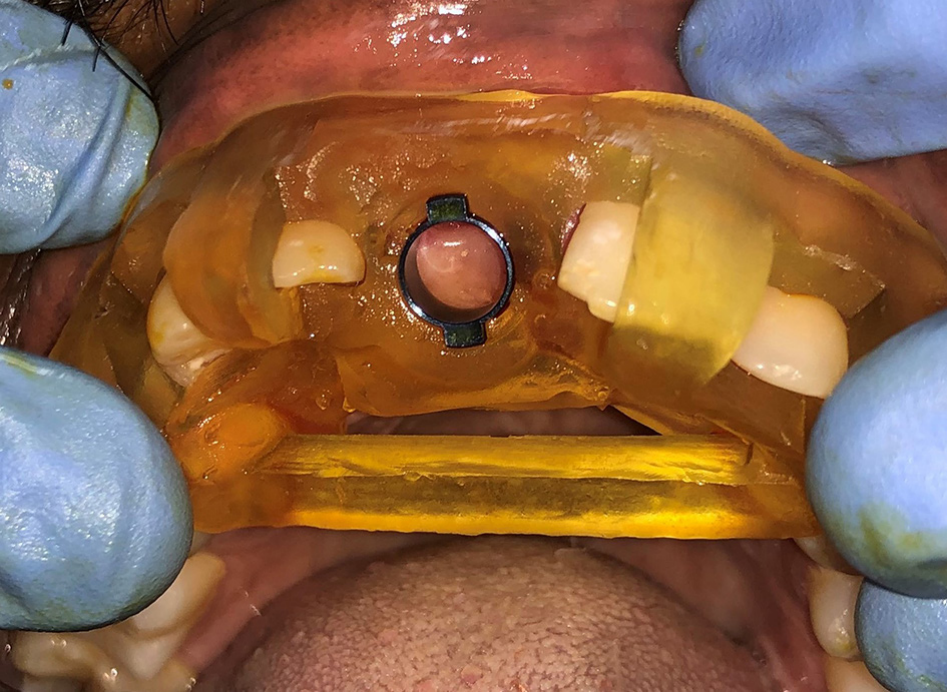
Surgical template placed and checked for case 1.

**Figure 6:**
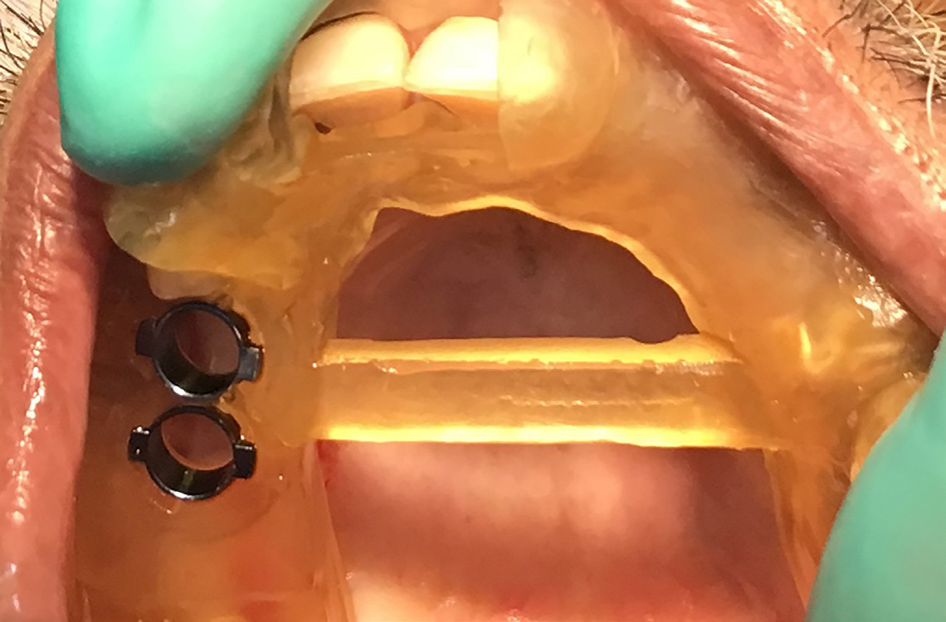
Surgical template placed and checked for case 2.

**Figure 7:**
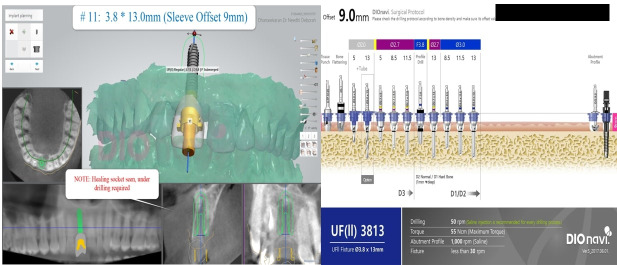
Planning information of case 1.

**Figure 8:**
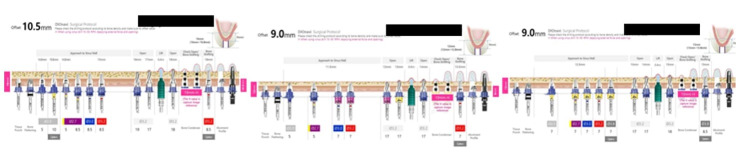
Planning information of case 2.

 The prepared guide is tooth-supported (Figures 5 & 6). Once the guide was checked on the patient and was found to be stable, the sequence of osteotomy was followed as per the approved 3D computerized planning (Figures 7 and 8). This sequence was based on the height and width of the implant to be placed in the remaining alveolar bone, the density of which was also assessed at the time of planning, along with the need for grafting and any sinus lift procedures as required. More than one drilling sequence is suggested in this procedure, depending on the quality of bone encountered during the osteotomy procedure. Following osteotomy, the implant was driven through the same provision in the guide. Finally, once the implant was inserted and secured, the guide was removed, and a hand wrench was used to further stabilize the implant in its assigned position. In the last step, a cover screw or a gingiva former was placed (as required). In our cases, a cover screw was placed in case 1 and gingiva formers in case 2. Following the osseointegration of implants, prosthetic crowns were advised.

###  Postoperative healing and further management

**Figure 9:**
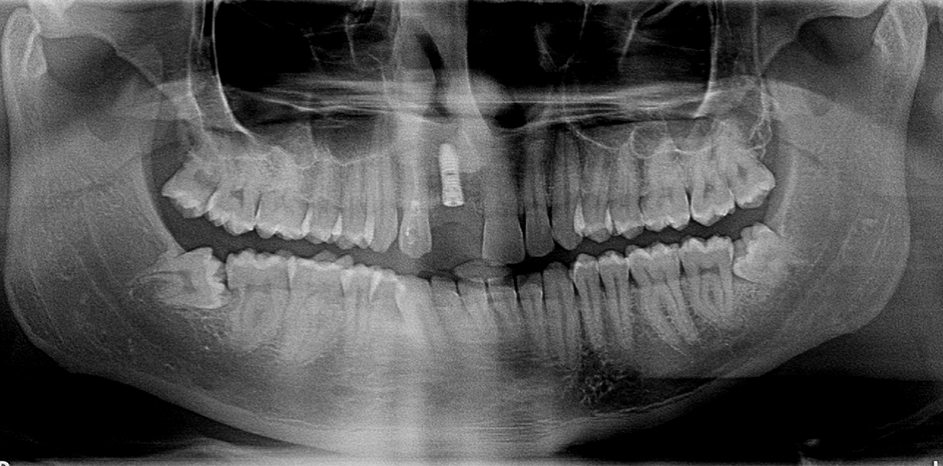
Postoperative panoramic radiograph after 5 months for osseointegration (case 1).

**Figure 10:**
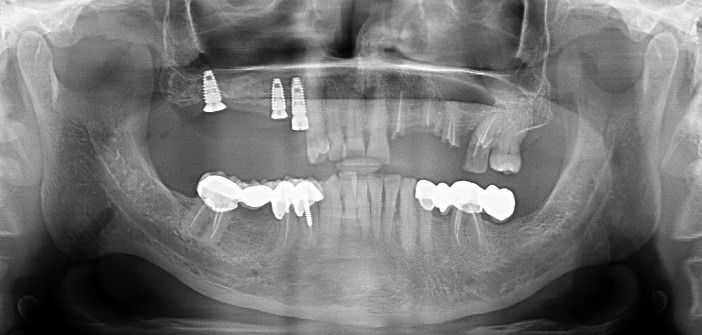
Postoperative panoramic radiograph after 5 months for osseointegration (case 2).

 The soft tissue healing was satisfactory after a week in both patients. The patients reported very mild discomfort for 1-2 days. Both patients were comfortable and satisfied with their pain-free experience and shorter treatment time during implant surgeries. Five months later, the treated sites were assessed in both cases. The healing was uneventful and satisfactory. Radiographs (Orthopantomographs- Pax-i3D Green SC; VATECH, Gyeonggi Province, South Korea) were obtained to assess osseointegration. As it was found to be sufficient (Figures 9 and 10), the patients were referred to the Department of Prosthodontics for further restorations.

 We have not included the information about restorations, as they are still being processed. The delay is due to the COVID-19 pandemic and restrictions. The patients have not been able to return regularly for subsequent visits and stages of crown placement. They are still under follow-up. We will update the journal once the crown placement and review adjustments are complete.

## Discussion

 The precise placement of implants, meeting all the prosthetic considerations, is as crucial as complete osseointegration is to meet the required functional and esthetic demands for the success of dental implants. Hence this approach of prosthetically driven implants, as seen in guided implant surgeries, is now becoming more important than the planning and design in implant dentistry.^5^ In the cases mentioned above, the static method of guided implant surgery was practiced, where 3D-printed surgical templates were used. The other method is the dynamic method in which a computer-guided navigation system helps the clinician in real-time during implant positioning through the visual imaging tools on a monitor. In the former, there are various types, namely, tooth-supported, bone- or mucosa-supported, with or without stabilization pins. In our cases, tooth-supported guides were used.^6^

 In guided implant surgery, the main advantage is its superior accuracy compared to implant placement using templates fabricated with standard radiographic tools (OPG) to decide the location of the implant arbitrarily. The other advantages of guided implant surgery are faster healing of soft tissues, minimum interference of blood supply, reduced bleeding, reduced surgical time, and greater patient comfort, as this is a flapless method. This minimally invasive approach increases the patient’s compliance and comfort during the treatment. It was also observed that placing implants by guided surgery required less time than conventional implant placement.^2,7-9^

 Regarding the accuracy of this technique, several studies have been carried out, and various conclusions have been reached. Some studies have debated this accuracy and predictability because of reports of differences in the planning and outcomes. Most discrepancies, especially angular deviations, were reported in the maxilla.^10^ Two reports concluded that there were apparent deviations, mainly because of difficulty in the stabilization of the surgical guide, especially in the mandible, due to inadequate surface area for support in comparison to the maxilla and the presence of a thick oral mucosa. Although these deviations may not have serious clinical implications, the authors suggested the need for a safety zone of 2 mm to avoid any injury to critical structures.^11,12^ However, in a randomized control study, the authors concluded that implant placement by guided implant surgery provided greater accuracy in a lateral direction compared to the use of conventional guides.^13^ Kernen et al.^8^ concluded that the rate of accuracy could be validated as high on in-vitro models, which is obviously a slightly different scenario than the clinical set-up, and attributed the inaccuracies to radiographic errors and the imperfect intraoral fit of the template along with patient- and operator-related factors.^14^ Cristache & Gurbanescu^15^ concluded that high accuracy was achieved when this technique was used. With varied conclusions made, it can be acknowledged that although the accuracy may not be 100%, it may be higher than conventional implant placement methods, especially in cases with adequate alveolar housing.^16^

 A systematic review concluded that there was a decrease in bone loss and effective re-growth of interdental papillary tissues, resulting in a more esthetic outcome in single implants using the guided implant technique.^17^ While there are significant benefits, there are also some drawbacks, such as lack of visibility, as the diameter of the drilling hole is just adequate for the passage of osteotomy drills.^18^ There are also reports of the possibility of removing a considerable amount of keratinized tissue around the dental implants.^7^ Being prosthetically driven, immediate loading was made possible, but some authors have stated that this is still in the preliminary state. However, there is another study with contradictory results, suggesting that it was feasible with high accuracy values.^19,20^ As with every new invention, this has its advantages and limitations; it would be wise to find ways to minimize the drawbacks because the advantages seem to outweigh the disadvantages.

## Conclusions

 Guided implant surgical approach was preferred because in case 1, the implant was to be placed in the esthetic zone with narrow bone width, and in case 2, the patient needed an indirect sinus lift procedure and being an older individual with systemic conditions, because we wanted to reduce the treatment time and postoperative complications. The patients were also anxiety-free and more cooperative during the treatment. The outcome of these procedures is satisfactory thus far, and the patients are under regular review.

## Authors’ contributions

 The case was diagnosed, planned, and supervised by SS. NDD was involved in the diagnosis and management of the case and manuscript preparation. The manuscript was edited by SS and RS and revised by RS and NDD. All authors have read and approved the final manuscript for submission.

## Funding

 There was no funding for this work.

## Availability of data

 Data concerning the cases mentioned above are available for reference.

## Consent to publication

 The patients whose data is reported in this paper gave written consents to publication. Consent to publication.

## Competing interests

 The authors declare no conflict(s) of interest related to the publication of this work.
